# Early effects of ventilatory rescue therapies on systemic and cerebral oxygenation in mechanically ventilated COVID-19 patients with acute respiratory distress syndrome: a prospective observational study

**DOI:** 10.1186/s13054-021-03537-1

**Published:** 2021-03-19

**Authors:** Chiara Robba, Lorenzo Ball, Denise Battaglini, Danilo Cardim, Emanuela Moncalvo, Iole Brunetti, Matteo Bassetti, Daniele R. Giacobbe, Antonio Vena, Nicolò Patroniti, Patricia R. M. Rocco, Basil F. Matta, Paolo Pelosi, collaborators, Pasquale Anania, Chiara Berri, Elena Ciaravolo, Chiara Dentone, Pietro Fiaschi, Paolo Frisoni, Angelo Gratarola, Laura Magnasco, Francesco Marramao, Marco Sottano, Lucia Taramasso, Fabio Tarantino, Gianluigi Zona

**Affiliations:** 1 Department of Surgical Sciences and Integrated Diagnostics (DISC), University of Genoa, Genoa, Italy; 2 IRCCS for Oncology and Neuroscience, Ospedale Policlinico San Martino, Genoa, Italy; 3 Department of Neurology, University of Texas, Austin, USA; 4 Department of Health Sciences (DISSAL), University of Genoa, Genoa , Italy; 5 Infectious Diseases Unit, IRCCS for Oncology and Neuroscience, Ospedale Policlinico San Martino, Genoa, Italy; 6 Laboratory of Pulmonary Investigation, Carlos Chagas Filho Institute of Biophysics, Federal University of Rio de Janeiro, Rio De Janeiro, Brazil; 7 Neurocritical Care Unit, Addenbrooke’s Hospital, Cambridge, UK; 8 Department of Neurosurgery, Ospedale Policlinico San Martino, Genoa, Italy

**Keywords:** Coronavirus, Cerebral oxygenation, Rescue therapies, Prone position, Recruitment maneuvers, Carbon dioxide removal

## Abstract

**Background:**

In COVID-19 patients with acute respiratory distress syndrome (ARDS), the effectiveness of ventilatory rescue strategies remains uncertain, with controversial efficacy on systemic oxygenation and no data available regarding cerebral oxygenation and hemodynamics.

**Methods:**

This is a prospective observational study conducted at San Martino Policlinico Hospital, Genoa, Italy. We included adult COVID-19 patients who underwent at least one of the following rescue therapies: recruitment maneuvers (RMs), prone positioning (PP), inhaled nitric oxide (iNO), and extracorporeal carbon dioxide (CO_2_) removal (ECCO_2_R). Arterial blood gas values (oxygen saturation [SpO_2_], partial pressure of oxygen [PaO_2_] and of carbon dioxide [PaCO_2_]) and cerebral oxygenation (rSO_2_) were analyzed before (T0) and after (T1) the use of any of the aforementioned rescue therapies. The primary aim was to assess the early effects of different ventilatory rescue therapies on systemic and cerebral oxygenation. The secondary aim was to evaluate the correlation between systemic and cerebral oxygenation in COVID-19 patients.

**Results:**

Forty-five rescue therapies were performed in 22 patients. The median [interquartile range] age of the population was 62 [57–69] years, and 18/22 [82%] were male. After RMs, no significant changes were observed in systemic PaO_2_ and PaCO_2_ values, but cerebral oxygenation decreased significantly (52 [51–54]% vs. 49 [47–50]%, *p* < 0.001). After PP, a significant increase was observed in PaO_2_ (from 62 [56–71] to 82 [76–87] mmHg, *p* = 0.005) and rSO_2_ (from 53 [52–54]% to 60 [59–64]%, *p* = 0.005). The use of iNO increased PaO_2_ (from 65 [67–73] to 72 [67–73] mmHg, *p* = 0.015) and rSO_2_ (from 53 [51–56]% to 57 [55–59]%, *p* = 0.007). The use of ECCO_2_R decreased PaO_2_ (from 75 [75–79] to 64 [60–70] mmHg, *p* = 0.009), with reduction of rSO_2_ values (59 [56–65]% vs. 56 [53–62]%, *p* = 0.002). In the whole population, a significant relationship was found between SpO_2_ and rSO_2_ (*R* = 0.62, *p* < 0.001) and between PaO_2_ and rSO_2_ (R0 0.54, *p* < 0.001).

**Conclusions:**

Rescue therapies exert specific pathophysiological mechanisms, resulting in different effects on systemic and cerebral oxygenation in critically ill COVID-19 patients with ARDS. Cerebral and systemic oxygenation are correlated. The choice of rescue strategy to be adopted should take into account both lung and brain needs.

*Registration* The study protocol was approved by the ethics review board (Comitato Etico Regione Liguria, protocol n. CER Liguria: 23/2020).

**Supplementary Information:**

The online version contains supplementary material available at 10.1186/s13054-021-03537-1.

## Introduction

In late December 2019, an outbreak of respiratory infection caused by a then-unknown virus was detected in Wuhan, China. Since then, severe acute respiratory syndrome coronavirus-2 (SARS-CoV-2) has spread worldwide, causing a pandemic of coronavirus disease 2019 (COVID-19) which overwhelmed intensive care units (ICUs) [1]. Although most patients with SARS-CoV-2 experience only mild symptoms such as fever and cough, a substantial number of patients develop severe hypoxemic respiratory failure, requiring intubation and mechanical ventilation [2, 3], with multiorgan failure in the most severe cases [4].

Recent publications have highlighted the specific features of COVID-19-associated acute respiratory distress syndrome (ARDS) [3, 5, 6], which make ventilatory management particularly challenging [7–10].

Patients with COVID-19-associated ARDS have a form of injury which, in many aspects, resembles ARDS unrelated to COVID-19 [11]. Adherence to evidence-based management has been recommended, including lung-protective mechanical ventilation and positive end-expiratory pressure (PEEP), as suggested by international guidelines for ARDS [12]. The value of other respiratory rescue therapies, such as recruitment maneuvers (RM), prone positioning (PP), inhaled nitric oxide (iNO), and carbon dioxide removal by ECCO_2_R or extracorporeal membrane oxygenation (ECMO), remains uncertain in this cohort of patients [13–16], with controversial efficacy concerning systemic oxygenation.

No data are available regarding the effect of these rescue therapies on cerebral hemodynamics, particularly on cerebral oxygenation. This latter point is of extreme importance, as neurological complications are common in mechanically ventilated critically ill patients with COVID-19 [17, 18] and may lead to impaired cerebral hemodynamics [17, 19]. Furthermore, respiratory rescue therapies may have detrimental effects on brain physiology, especially in the early phases after application, when the major hemodynamic and respiratory changes occur; therefore, their application in brain-injured patients outside of the COVID-19 pandemic is currently debated [20–22].

We hypothesized that each rescue strategy would have different effects on respiratory and cerebral oxygenation. Thus, the choice of ventilatory rescue therapy should take into account both lung and cerebral needs.

A prospective observational study was conducted to assess the early effects of different ventilatory rescue therapies currently used in ICU (RMs, PP, iNO or ECCO_2_R) on systemic and cerebral oxygenation in mechanically ventilated patients with COVID-19-associated ARDS. For this purpose, arterial blood gases and systemic and cerebral hemodynamics were analyzed. The correlation between systemic and cerebral oxygenation in the whole population was also assessed before and after rescue therapies.

## Methods

### Study design

This study followed the “Strengthening the Reporting of Observational Studies in Epidemiology (STROBE)” statement guidelines for observational cohort studies (Additional file 1) [23]. This prospective, single-center observational study was conducted in a university-affiliated hospital in Genoa, northern Italy (Ospedale Policlinico San Martino, IRCCS for Oncology and Neuroscience). The study protocol was approved by the local ethics review board (Comitato Etico Regione Liguria, protocol n. CER Liguria: 23/2020). Written consent was obtained from next of kin, as patients were unconscious at the time of inclusion.

### Study population

This study included consecutive critically ill patients with COVID-19, as confirmed by SARS-CoV2 polymerase chain reaction on nasopharyngeal swab specimens, admitted to our ICU during the second wave of the SARS-CoV-2 pandemic in Italy (from October 1, 2020, to December 15, 2020).

Further inclusion criteria were adult age (≥ 18 years old), requiring intubation and mechanical ventilation, and acute onset of ARDS, as defined by the Berlin criteria [24], which included new or worsening respiratory symptoms due to SARS-CoV-2 infection with hypoxemia (defined by a ratio between partial pressure of oxygen in arterial blood [PaO_2_] and fraction of inspired oxygen [PaO_2_/FiO_2_] ≤ 300 mmHg on positive end-expiratory pressure [PEEP] ≥ 5 cmH_2_O, regardless of FiO_2_; presence of bilateral pulmonary infiltrates on chest imaging [X-ray or computed tomography]; and absence of left atrial hypertension or no clinical signs of left heart failure), who required one or more ventilatory rescue therapies according to clinical needs (RMs, PP, iNO, ECCO_2_R), and who contemporarily underwent noninvasive multimodal neuromonitoring, including cerebral oxygenation using near-infrared spectroscopy (NIRS) and transcranial Doppler (TCD) as per our local clinical practice.

The exclusion criteria were non-confirmed SARS-CoV-2 infection according to WHO guidance [25], patients with no data at baseline or who did not undergo any type of rescue therapies or neuromonitoring, or those in which TCD or NIRS could not be performed (absence of temporal window or space in the forehead for NIRS sensor positioning).

### Data collection

Demographic, epidemiologic, and clinical data were collected from electronic medical records, both at admission to the ICU and on the day when each rescue therapy was performed. Data from patients’ electronic medical records were reviewed and collected by physicians trained in critical care. Patients’ confidentiality was protected by assigning a de-identified patient code.

### General monitoring data

Recorded data included admission demographics such as age, gender, Sequential Organ Failure Assessment (SOFA), body mass index (BMI), comorbidities (hypertension, diabetes mellitus, chronic kidney injury, chronic respiratory disease, previous neurological disease, liver failure, chronic cardiac disease), vital signs such as mean arterial pressure (MAP), heart rate (HR), laboratory parameters such as blood test, D-dimer, C-reactive protein (CRP), procalcitonin (PCT), creatinine, hemoglobin (Hb) and ventilatory parameters such as tidal volume (V_T_), FiO_2_, respiratory rate (RR), PEEP, plateau pressure (Pplat), respiratory system compliance (Crs), ICU length of stay (LOS), and mortality.

Ventilatory parameters such as PEEP, Pplat, Crs, V_T_, FiO_2_, saturation of oxygen (SpO_2_), pHa, PaO_2_, partial pressure of carbon dioxide (PaCO_2_), systemic (MAP, HR) and neuromonitoring parameters (TCD and NIRS-derived indices) were obtained before (T0) and after (T1) the application of any type of rescue therapy.

### Ventilator management and rescue therapies

Patients were sedated with propofol (or midazolam) and fentanyl and paralyzed with a continuous infusion of cisatracurium besilate. They were ventilated in pressure-controlled mode (P-CMV), aiming to maintain PPlat < 28 cmH_2_O, using a V_T_ of 4–8 mL/kg of predicted body weight (PBW); FiO_2_ and PEEP were titrated in order to achieve SpO_2_ 88–92%, and RR to aim for PaCO_2_ = 35–45 mmHg or allow permissive hypercapnia as long as pHa was maintained in range.

The decision to start any type of rescue therapy was related to the clinician’s assessment and judgment. In our institution, the use of rescue therapies (RM, PP, iNO) was considered when patients presented severe ARDS with PaO_2_/FiO_2_ values < 100 for more than 6 h with worsening clinical trajectory [26].

ECCO_2_R was considered when pHa was below 7.3 and/or PaCO_2_ higher than 70 mmHg, with Pplat higher than 27 cmH2O, not responsive to conventional treatment.

As for our local protocol, after optimizing lung protective strategies, we progressively increased the FiO_2_ to 100% before starting any type of rescue therapy. From T0 and T1, FiO_2_ was not modified. In case of refractory hypoxemia, RMs and/or iNO and prone positioning were used. Recruitment maneuvers were used when the patients were considered potentially PEEP responders according to respiratory system mechanics and computed tomography (CT) findings. In the presence of posterior atelectasis at the lung CT, prone positioning was the treatment of choice. Finally, iNO was considered in cases of refractory hypoxemia with clinical suspect on echocardiography of pulmonary hypertension and CT findings.

#### Recruitment maneuvers

Recruitment maneuvers were applied using an escalating PEEP strategy, as is common practice at in our institution. For PEEP titration, V_T_ was kept constant and PEEP was increased up to maximal inspiratory pressure 35–40 cmH_2_O for 30 s (five breaths at each PEEP level) followed by decremental PEEP titration according to SpO_2_, respiratory system mechanical properties and hemodynamic parameters. T1 measurements were taken 5–10 min after the RM.

#### Prone positioning

Patients were carefully turned from the supine to prone position by a team of 4 staff members (3 staff nurses and 1 physicians); 2 on each side and 1 (the anesthetist) controlling head and airways and coordinating the procedure.

In the PP, we limited the shoulder abduction to < 90 degrees to avoid overstretching of the brachial plexus. We placed the forearm in a neutral position to minimize the direct pressure on the ulnar nerve at the elbow and applied soft padding under the elbows, chest, and pelvis. The potential for increased intrathoracic pressure caused by increased abdominal pressure in the prone position was minimized by using foam padding to limit abdominal compression. The head was put in neutral position on an open soft head ring (Horseshoe Head Pad—High—Adult Size) to avoid any direct pressure to the eyes, nose, and mouth. T1 measurements were taken 30 min–1 h after patient positioning.

#### Inhaled nitric oxide

Nitric oxide gas was provided through the breathing circuit (Maquet-Kinox, Healthcare, Canada 2020) at a test concentration of 20 ppm. After assessing the response of systemic and cerebral oxygenation to iNO (from T0 and T1 after 1 h) [27], iNO was titrated according to patients’ needs and arterial blood gases.

#### *Extracorporeal CO*_*2*_* removal*

ECCO_2_R was started in case of refractory hypercapnia with decompensated pHa and was provided using two methods using systemic heparin anticoagulation: (1) using a dedicated or pump-driven venovenous (EstorFlow®, Estor, Milan, Italy) ECCO_2_ removal device. ECCO_2_R was commenced at a blood flow of 200 mL/min and air flow of 10–12 l/min, and then blood and gas flows were titrated according to patients’ response and arterial blood gases values. (2) a polymethylpentene, hollow-fiber, gas-exchanger membrane (multiECCO_2_R; Eurosets, Medolla, Italy), a labeled and certified European device to be used in conjunction with multiFiltrate continuous renal replacement therapy (CRRT) platforms (Fresenius Medical Care, Bad Homburg, Germany) for combined respiratory and renal support. ECCO_2_R + CRRT was commenced at a blood flow of 200–400 mL/min, and continuous venovenous hemodialysis (CVVHD) was delivered with an effluent dose of 25 mL/kg/h; blood flow was increased stepwise according to the patient response. T1 measurements were taken after 20 min from ECCO_2_R initiation.

### Neuromonitoring data

#### Cerebral oxygenation

Continuous regional cerebral oxygen saturation was obtained using a Masimo Root monitor® (USA) with bilateral sensors applied to the frontotemporal area. Final cerebral oxygenation was calculated as the mean between the right and left frontotemporal sensors. The Masimo tool is able to noninvasively estimate different innovative NIRS-derived parameters (Additional file 1: Fig. S1):

- rSO_2_: total value of regional cerebral oxygen saturation.
- Variation of O_2_Hbi (ΔO_2_Hbi): an index associated with variation of the oxygenated component of the Hb of the total calculation of rSO_2_, thus representing changes in the arterial component of rSO_2_.
- Variation of HHbi (ΔHHbi): an index associated with variation of the deoxygenated component of Hb within the total calculation of rSO_2_, thus representing changes in the venous component of rSO_2_.
- Variation of cHbi (ΔcHbi) is the sum of the values of ΔO_2_Hbi e ΔHHbi to calculate the value rSO_2_ (ΔcHbi = ΔHHbi + ΔO_2_Hbi)
- Variation of SpO_2_–rSO_2_: difference between the value of SpO_2_ and rSO_2_

#### Noninvasive intracranial pressure assessment

Noninvasive ICP (nICP) was measured using the transcranial color duplex Doppler technique (Philips Bothwell 98021®, USA), with a low-frequency (2 MHz) echo graphic micro-convex probe to investigate intracranial vessels. The temporal window was used to assess bilaterally the proximal part of the mean cerebral artery (MCA) [28, 29]. Systolic, diastolic, and mean flow velocities (FVs, FVd, and FVm, respectively) were obtained bilaterally from the MCA, and nICP is calculated according to the formula [30]:

$$\begin{aligned}{\text{nICP}}&={\text{MAP}}-{\text{noninvasive cerebral perfusion pressure(nCPP) }}\\ &\qquad {\text{nCPP was calculated as MAP}} \times {\text{(FVd/FVm)}}+14.\end{aligned}$$

The final nICP was calculated as the mean of the right and left nICP in both the MCAs.

### Statistical analysis

No data on arterial and cerebral oxygenation after rescue therapies are available in COVID-19 patients, and therefore, a formal a priori sample size calculation was not feasible. However, the achieved sample size was comparable to other physiologic studies in the field [31–33]. For the two rescue therapies for which we observed the highest impact on arterial and cerebral oxygenation (RMs and PP), the achieved sample size resulted in a power (1-β) above 90%, assuming an intrasubject correlation of *R* = 0.5.

The Shapiro–Wilk test was used to test the normality of the distribution of the results. Data are reported as median and interquartile range [IQR = 25th–75th percentiles], if not otherwise specified.

Comparisons between different variables at T0 and T1 were made by repeated measures *t* test, while non-normally distributed variables were compared by Wilcoxon signed-rank test.

The correlations between cerebral and systemic oxygenation were verified. Correlations with repeated measurements were computed according to the Bland and Altman method [34, 35]. All statistical analyses were performed using SPSS 21® (IBM corp., US) and RStudio software (version 4.0.3). A *p* < 0.05 was considered statistically significant.

## Results

### Baseline characteristics

Thirty-eight patients with COVID-19-associated ARDS were admitted to the ICU during the study period. Among these, 22 patients received at least one or more rescue therapies and were included in the final analysis. A total of 45 rescue therapies were used, and measurements before and after rescue therapies (at T0 and T1) were taken from each patient.

Demographic and clinical characteristics of the population included in our study are presented in Additional file 1: Table S1. The median age was 62 years [IQR = 57–69], and 18/22 (82%) were male. On ICU admission, the median PaO_2_/FiO_2_ was 81 mmHg [IQR = 65–82.5]. Rescue therapies included 22 RMs, 9 iNO administrations, 10 prone positionings, and 4 ECCO_2_R or respiratory dialysis applications.

### Effect of rescue therapies in the overall population

Considering the overall population, at T0, the median PEEP was 12 (IQR = [11–13]) cmH_2_0, median Pplat 29 [28–30] cmH_2_0, and median V_T_ 5.7 [5.5–6.2] ml/predicted body weight (PBW); the median baseline PaO_2_ was 66 [62–72] mmHg, PaCO_2_ 54 [48–64] mmHg, pHa 7.37 [7.33–7.41], median MAP 72 [68–76.5] mmHg, and median HR was 75 [65–88.5] beats per minute (Table 1).

**Table 1:** Ventilator settings, neuromonitoring values, and hemodynamic data at time points T0 (before rescue therapy) and T1 (after rescue therapy)

Parameter	All (*N* = 45)			RM (*N* = 22)RM (*N* = 22)			Prone position (*N* = 10)		
	T0	T1	*p* value	T0	T1	*p* value	T0	T1	*p* value
*Primary outcome*									
rSO_2_ (%)	53 [51–55]	52 [49–59]	0.692	52 [51–54]	48.5 [47–50]	< 0.0001*	53 [52–54]	59.5 [59–64]	0.005*
PaO_2 /_FiO_2_	66 [62–72]	71 [67–74]	0.002*	66 [62–72]	67.5 [63–72]	0.189	62 [56–71]	81.5 [76–87]	0.005*
SpO_2_ (%)	88 [87–90]	91 [89–92]	< 0.0001*	88 [87–89]	89 [88–91]	0.072	87 [86–88]	93 [89–96]	0.005*
*Ventilator settings*									
PEEP (cmH_2_O)	12 [11–13]	12 [11–13]	0.169	12 [11–13]	12 [10–13]	0.157	12 [11–13]	12 [10–13]	0.317
Pplat (cmH_2_O)	29 [28–30]	29 [28–30]	0.698	29 [28–30]	29 [28–30]	1.000	29 [28–30]	29 [28–30]	1.000
VT/PBW (mL/Kg)	5.7 [5.5–6.2]	5.9 [4.9–6.9]	0.086	6.7 [6–7.1]	6.3 [5.4–6.6]	0.001*	5.4 [5.1–6.1]	6.3 [6–7.1]	0.005*
RR (n/min)	26 [24.5–27.5]	26 [24.5–28]	0.026*	25 [24–26]	25 [24–26]	0.109	27.5 [26–28]	27.5 [26–28]	1.000
Crs (mL/cmH_2_O)	20.1 [18.2–24.7]	19.8 [17.8–23.5]	0.615	21.6 [19.4–25.2]	20.1 [19.6–24.5]	0.200	14.9 [13.4–18.6]	23.4 [19.5–26.4]	0.005*
*Neuromonitoring*									
ΔcHbi	4.3 [3–6]	4.7 [2.6–7.7]	0.157	5 [3.7–6.1]	3.2 [2.5–4.6]	< 0.0001*	4.5 [2.4–6.5]	10.2 [8.9–12.4]	0.005*
ΔO2Hbi	3.2 [2.7–4.2]	2.1 [1.4–5.3]	0.991	3.7 [2.9–4.4]	1.7 [1.3–2.1]	< 0.0001*	3.5 [2.1–4.6]	8 [6.3–8.8]	0.005*
ΔHHbi	0.9 [0.3–1.8]	2.1 [1.2–2.8]	< 0.0001*	1.1 [0.8–1.8]	1.3 [1.1–2.2]	0.001*	1 [0.2–1.9]	2.3 [2.1–3.9]	0.005*
nICP (mmHg)	17 [15.5–19]	21 [17–26]	< 0.0001*	17.5 [15–19]	26 [24–28]	< 0.0001*	16 [15–18]	18 [16–20]	0.016*
nCPP (mmHg)	55 [51–60]	50 [39–59]	0.001*	54 [50–62]	39 [35–44]	< 0.0001*	55.5 [53–58]	60.5 [59–63]	0.011*
ΔSpO_2_rSO_2_ (%)	36 [37–37	38 [33–41]	0.001*	36 [35–37]	41 [38–43]	< 0.0001*	33.5 [32–35]	31.5 [30–33.5]	0.134
*Other arterial blood gas values*									
pHa	7.37 [7.33–7.41]	7.38 [7.35–7.41]	0.017*	7.38 [7.34–7.41]	7.38 [7.35–7.42]	0.466	7.37 [7.34–7.41]	7.41 [7.38–7.43]	0.024*
PaCO_2_ (mmHg)	54 [48–64]	53 [47–63]	< 0.0001*	51 [46–56]	50 [45–56]	0.108	53.5 [47–71]	53.3 [47–69]	0.021*
*Hemodynamics*									
Hb (mg/dL)	8.5 [8.1–8.8]	8.3 [8.1–8.7]	0.119	8.6 [8.2–8.8]	8.4 [8.3–8.8]	0.343	8.5 [8.1–8.9]	8.3 [8.1–8.7]	0.321
MAP (mmHg)	72 [68–76.5]	71 [65–76]	0.013*	71.5 [67–76]	64 [59–68]	< 0.0001*	72.5 [68–75]	78.5 [71–84]	0.005*
HR (n/min)	75 [65–88.5]	77 [68–94]	0.015*	77.5 [68–89]	79 [69–91]	0.009*	77 [75–85]	78 [72–83]	0.878

Parameter	iNO (*N* = 9)			ECCO_2_R (*N* = 4)		
	T0	T1	*p* value	T0	T1	*p* value
*Primary outcome*						
rSO_2_ (%)	53 [51–56]	57 [55–59]	0.007*	59 [55.5–64.5]	56 [52.5–62]	0.002*
PaO_2 /_FiO_2_	65 [67–73]	72 [67–73]	0.015*	75 [74.5–78.5]	63.5 [60–69.5]	0.009*
SpO_2_ (%)	89 [88–90]	92 [91–93]	0.016*	93 [91.5–94]	89.5 [87.5–91]	0.01*
*Ventilator settings*						
PEEP (cmH_2_O)	12 [11–14]	12 [10–13]	0.025*	12 [1113]	10.5 [10–11]	0.103
Pplat (cmH_2_O)	29 [28–29]	29 [28–29]	1.000	29.5 [28–30.5]	29.5 [28–30.5]	0.543
VT/PBW (mL/Kg)	5.7 [5.2–7.4]	5.9 [5.3–7.2]	0.441	5.7 [3–6.7]	4.3 [3.8–6]	0.068
RR (n/min)	25 [20–26]	26 [22–26]	0.102	29 [27–30]	29 [27–30]	0.225
Crs (mL/cmH_2_O)	20.8 [19.3–26.5]	16.7 [19.6– 26.6]	0.086	17 [15.9–18.9]	13.5 [11.3–15.7]	0.465
*Neuromonitoring*						
ΔcHbi	3.1 [3–4.3]	7.1 [7–8.1]	0.008*	3.6 [2.7–4.2]	1.3 [0.7–1.9]	0.002*
ΔO2Hbi	2.8 [2.6–3.7]	4.7 [4.1–5.2]	0.008*	3 [2.3–3.4]	0.6 [0.2–1.2]	0.002*
ΔHHbi	0.4 [0.3–1.2]	2.8 [1.9–2.9]	0.008*	0.6 [0.3–0.8]	0.7 [0.4–0.8]	0.182
nICP (mmHg)	18 [17–19]	17 [16–18]	0.033*	22.5 [19–26]	18.5 [15–23]	0.006*
nCPP (mmHg)	59 [54–60]	59 [53–60]	0.395	50.5 [50–52]	53 [52–54.5]	0.059
ΔSpO_2_rSO_2_ (%)	36 [34–37]	35 [33––37]	0.320	34 [29.5–36]	33.5 [29–35]	0.058
*Other arterial blood gas values*						
pHa	7.36 [7.35–7.38]	7.38 [7.36–7.39]	0.031*	7.21 [7.19–7.26]	7.36 [7.34–7.37]	0.008*
PaCO_2_ (mmHg)	54 [48–68]	55[48–67]	0.058	100 [99–100]	72 [70–73]	< 0.0001*
*Hemodynamics*						
Hb (mg/dL)	8.1 [7.9–8.3]	8.3 [7.7–8.3]	0.432	8.1 [7.8–8.5]	8.2 [7.9–8.5]	0.314
MAP (mmHg)	76 [71–76]	75 [70–77]	0.248	73 [69–78]	71.5 [67–77.5]	0.141
HR (n/min)	69 [64–75]	67 [65–89]	0.917	65.5 [65–71.5]	73 [69.5–78]	0.049*

Values are presented as median and Interquartile range if not otherwise specified

*ECCO*_*2*_*R* extracorporeal carbon dioxide removal, *RM* recruitment maneuvers, *iNO* inhaled nitric oxide, *PaO*_*2*_ partial pressure of oxygen, *FiO*_*2*_ inspired fraction of oxygen ratio, *SpO*_*2*_ oxygen saturation, *PEEP* positive end-expiratory pressure, *Pplat* plateau pressure, *VT* tidal volume, *PBW* predicted body weight, *RR* respiratory rate, *Crs* respiratory system compliance, *rSO*_*2*_ cerebral oxygenation saturation, *nICP* noninvasive intracranial pressure, *nCPP* noninvasive cerebral perfusion pressure

DeltaO_2_Hbi (ΔO_2_Hbi), change in the oxygenated component of hemoglobin (Hb); delta HHbi (ΔHHbi), change in the deoxygenated component of Hb; delta cHbi (ΔcHbi), sum of the values of ΔO_2_Hbi; ΔcHbi = ΔHHbi + ΔO_2_Hbi; ΔSpO_2_–rSO_2_, difference between the value of SpO_2_ and rSO_2_; *N* number

The baseline median rSO_2_ was 53% [51–55], with ΔcHbi 4.3 [3–6], ΔHHbi 0.9 [0.3–1.8] and ΔO_2_Hbi 3.2 [2.7–4.2]; the nICP was 17 [15.5–19].

At T1, SpO_2_, PaO_2_, and nICP values were significantly increased compared to T0 (88 [87–90] vs. 91% [89–92], *p* < 0.001; 66 [62–72] vs. 71 [67–74] mmHg; *p* = 0.002; 17 [15.5–19] vs. 21 [17–26] mmHg, *p* < 0.001, respectively), whereas PaCO_2_ was significantly lower compared to T0 (median = 54 [48–64] mmHg vs. 53 [47–63]; *p* < 0.001). No differences in rSO_2_ were observed between T0 and T1 (54 [48–64] vs. 53 [47–63] %, *p* = 0.692) (Table 1). From T0 to T1, nCPP was reduced (55 [51–60] vs. 50 [39–59], *p* = 0.001). Additional file 1: Table S2 presents the effect of rescue therapies according to subgroups with low or high PaO_2_, dichotomized according to the median value of PaO_2_.

### Correlations between systemic and cerebral oxygenation in the overall population

**Fig. 1:**
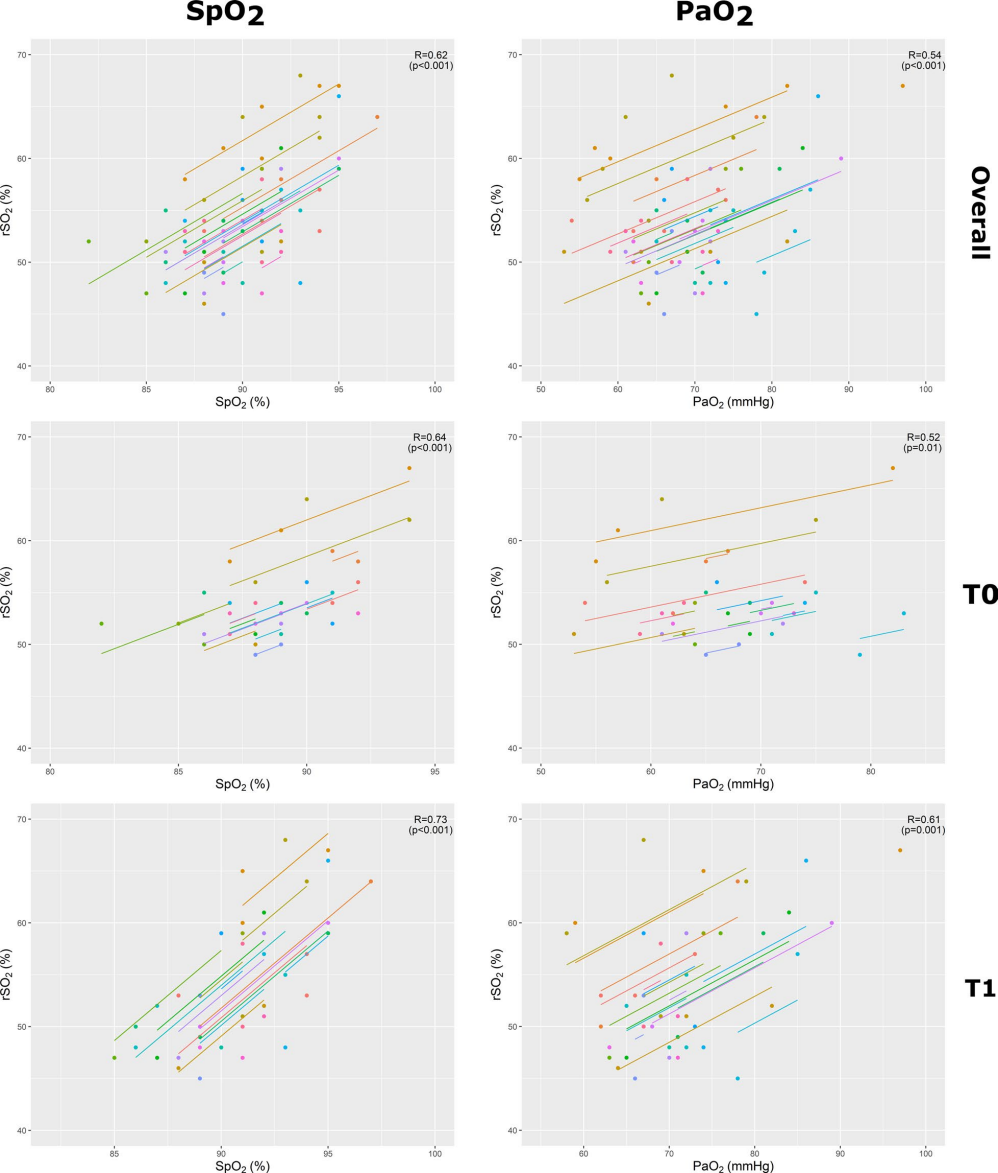
Scatterplots showing the linear association and correlation (*R*) between systemic oxygen saturation (SpO_2_) (left panel) and partial pressure of oxygen (PaO_2_) (right panel) versus cerebral oxygenation (rSO_2_) at different study timepoints. Repeated measurements for each patient are plotted in the same color pattern. Linear regression lines are correspondent to repeated measurements within patients

*C*onsidering the entire data (T0 + T1 = 90 measurements), a statistically significant correlation between SpO_2_ and rSO_2_ values was observed overall (*r* = 0.62, *p* < 0.001), at T0 (*r* = 0.64, *p* < 0.001), and T1 (*r* = 0.73, *p* < 0.001) (Fig. 1). PaO_2_ and rSO_2_ were also overall correlated (*r* = 0.54, *p* < 0.001.), at T0 ((*r* = 0.52, *p* = 0.01) and T1 (*r* = 0.61, *p* = 0.001) (Fig. 1). We also found a significant correlation between the changes in rSO_2_ and in SpO_2_, PaO_2_, and rSO_2_ (Additional file 1: Fig. S2). Finally, a statistically significant correlation was found between PaCO_2_ and rSO_2_ (T0 *r* = 0.31, *p* = 0.038; T1 *r* = 0.269, *p* = 0.074; overall values *r* = 0.315, *p* = 0.002), and between MAP and rSO_2_ (T0 *r* = 0.262, *p* = 0.082; T1 *r* = 0.609, *p* = 0.001; overall values *r* = 0.357, *p* = 0.001).

### Specific subgroups

**Fig. 2:**
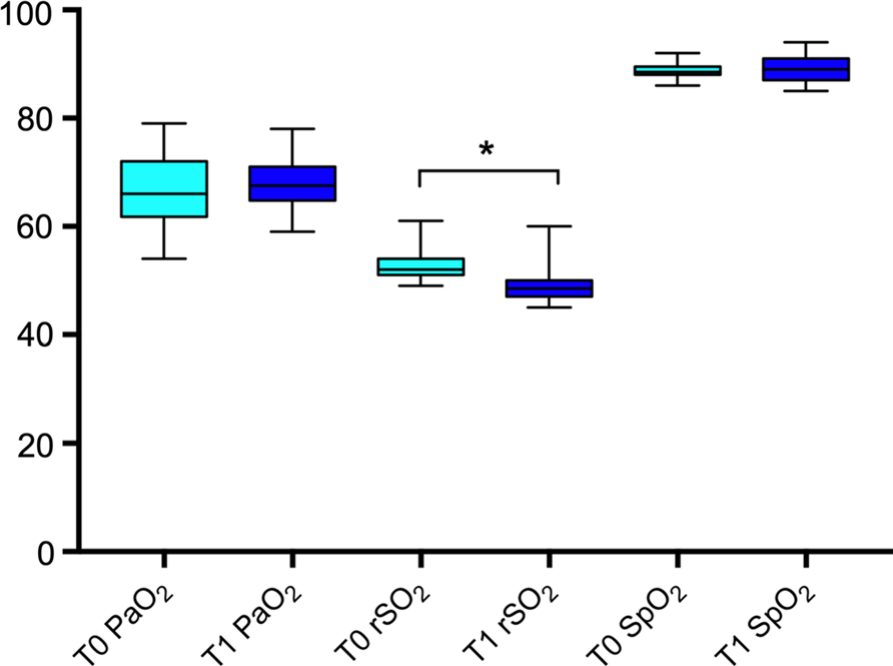
Boxplots representing the effect of recruitment maneuvers (RMs) on partial pressure of oxygen (PaO_2_), cerebral oxygenation (rSO_2_), and oxygen saturation (SpO_2_) from baseline, T0 (pre), and after RMs, T1 (post). Values are presented as median and interquartile range

Twenty-two patients underwent RMs, after which no significant changes were observed in PaO_2_ and PaCO_2_ values (Table 1). At T1, values of cerebral oxygenation (both arterial and venous components) were decreased (rSO_2_ 52 [51–54] vs. 48.5% [47–50], *p* < 0.001), whereas nICP increased significantly compared to T0 (17.5 [15–19] vs. 26 [24–28] mmHg, *p* < 0.001). Furthermore, MAP and nCPP were decreased after RM application (71.5 [67–76] vs. 64 [59–68] mmHg, *p* < 0.001 and 54 [50–62] vs. 39 [35–44] mmHg, *p* < 0.001, respectively) (Fig. 2).

**Fig. 3:**
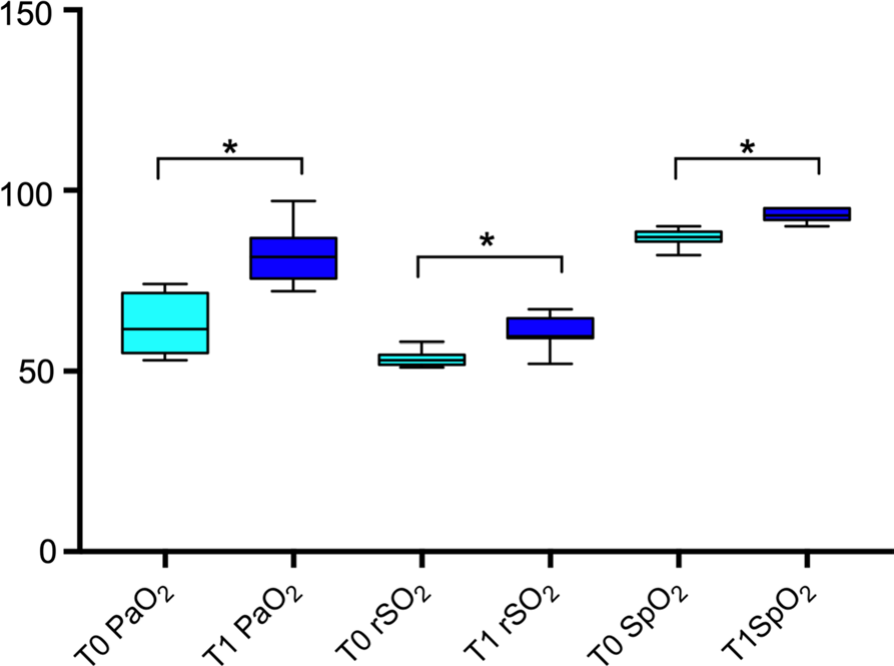
Boxplots representing the effect of prone positioning on partial pressure of oxygen (PaO_2_), cerebral oxygenation (rSO_2_), and oxygen saturation (SpO_2_), from baseline, T0 (pre), and after prone positioning, T1 (post). Values are presented as median and interquartile range

After prone positioning (*N* = 10), systemic and cerebral oxygenation increased significantly (PaO_2_ = 62 [56–71] vs. 81.5 [76–87]mmHg, *p* = 0.005; rSO_2_ = 53 [52–54]% vs. 59.5 [59–64]%, *p* = 0.005), with a slight increase in nICP (16 [15–18] vs. 18 [16–20] mmHg, *p* = 0.016), MAP (72.5 [68–75] vs. 78.5 [71–84]mmHg, *p* = 0.005), and nCPP (55.5 [53–58] vs. 60.5 [59–63]mmHg, *p* = 0.011) (Fig. 3).

**Fig. 4:**
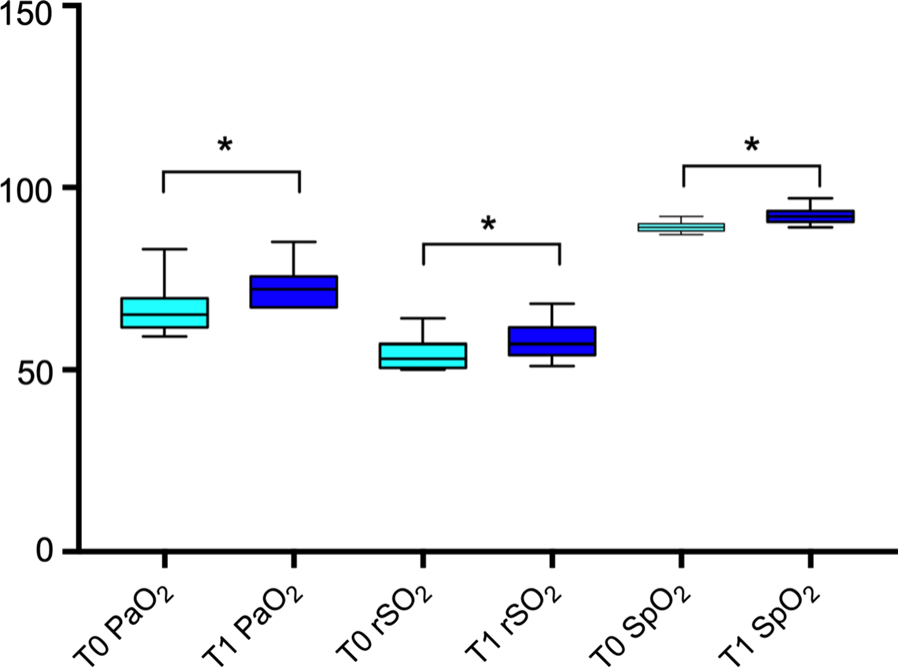
Boxplots representing the effect of inhaled nitric oxide (iNO) on partial pressure of oxygen (PaO_2_), cerebral oxygenation (rSO_2_), and oxygen saturation (SpO_2_) from baseline, T0 (pre), and after iNO, T1 (post). Values are presented as median and Interquartile range

After iNO, both systemic and cerebral oxygenation values increased (65 [67–73] vs. 72 [67–73] mmHg, *p* = 0.015, and 53 [51–56]% vs. 57 [55–59]%, *p* = 0.007, respectively), with no effect on MAP and nCPP and a slight reduction of nICP (18 [17–19] vs. 17 [16–18] mmHg, *p* = 0.033) (Fig. 4).

**Fig. 5:**
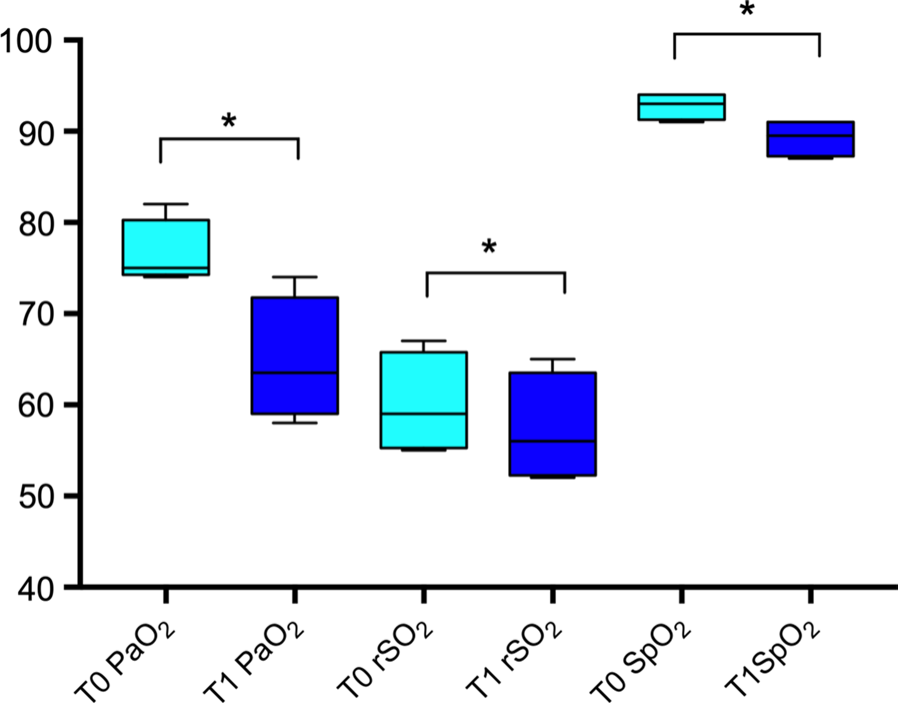
Boxplots representing the effect of ECCO_2_R or respiratory dialysis on partial pressure of oxygen (PaO_2_), cerebral oxygenation (rSO_2_), and oxygen saturation (SpO_2_) from baseline, T0 (pre), and after CO_2_ removal, T1 (post). Values are presented as median and interquartile range

Finally, ECCO_2_R was used in four patients. The use of CO_2_ removal resulted in a decrease in PaO_2_ (75 [74.5–78.5] vs. 63.5 [60–69.5] mmHg, *p* = 0.009), SpO_2_ (93 [87.5–91] vs. 89.5 [87.5–91]%), PaCO_2_ (100 [9–100] vs. 72 [70–73] mmHg, *p* < 0.001), and rSO_2_ (59 [55.5–64.5] vs. 56 [52.5–62]%, *p* = 0.002), and in particular, in the oxygenated component of ΔcHbi (ΔO_2_Hbi = 3.6 [2.7–4.2] vs. 1.3 [0.7–1.9], *p* = 0.002) (Table 1; Fig. 5).

## Discussion

In the present study, we investigated the early effects of different types of rescue therapies on systemic and cerebral oxygenation in patients with COVID-19-associated ARDS. We found that iNO and prone positioning improved systemic and cerebral oxygenation; RMs did not improve systemic oxygenation, but worsened rSO_2_; respiratory dialysis/ECCO_2_R reduced both systemic and cerebral oxygenation, and in the whole population, a significant correlation was found between SpO_2_ and rSO_2_, and between rSO_2_ and PaO_2 _.

To our knowledge, this is the first study investigating the early effects of rescue therapies on systemic and cerebral oxygenation and their correlation in critically ill patients with COVID-19-associated ARDS. The use of multimodal neuromonitoring, including new indices such as ΔHHbi + ΔO_2_Hbi, enabled us to better investigate the specific consequences of each ventilatory rescue strategy for brain and lung function. This is particularly important, especially in the early phases after rescue therapies application, when most of the effects on cerebral physiology are mainly acting.

The lung and brain are important organs to be monitored. COVID-19 patients often present with severe hypoxemia not responsive to conventional treatment and at the same time are at high risk of neurological complications [17, 36]. In this context, the role of rescue therapies generally used to improve oxygenation and outcomes in conventional severe ARDS [37–39] has not been completely elucidated in COVID-19 [14, 40–43].

In a recent prospective physiological study [31] where a two-step positive end-expiratory pressure trial with change of 10 cmH_2_O was applied, potential for lung recruitment was found to vary widely among patients. Similarly, the efficacy of iNO has not been completely defined in COVID-19 patients; although iNO can significantly improve oxygenation, probably helping in redistribution of pulmonary flow [14], its effect on oxygenation is inconsistent among studies [44]. Prone positioning has been increasingly used during the pandemic [30, 41, 45], and preliminary reports suggest a beneficial effect of this maneuver on the PaO_2_/F_i_O_2_ ratio. Finally, few case reports [13] are available regarding the use of respiratory dialysis/ECCO_2_R in this population as an adjuvant therapy to limit further ventilator-induced lung injury. The potential harmful effects of these therapies on cerebral hemodynamics have not been investigated. Although COVID-19 patients are not primarily brain-injured, a significant proportion of them experience neurological complications [36, 46, 47]; the pathophysiology of such complications in this cohort of patients is complex and probably multifactorial, including different mechanisms such as viral neurotropism, hypercoagulability, and brain–lung crosstalk [48], with cerebral hypoxemia [17, 19] consequent to severe respiratory failure. The use of rescue therapies can further aggravate the delicate relationship between the brain and the lungs in these patients [49–54]. The use of RM and prone positioning can potentially increase intrathoracic pressure and therefore ICP [28], while extracorporeal systems such as respiratory dialysis/ECCO_2_ removal or ECMO may potentially increase the risk for intracerebral hemorrhage [55].

The concept of protective ventilation and the use of rescue therapies is slowly gaining interest even in this population, although evidence is still lacking [20, 56, 57]; small studies and a recent expert consensus on mechanical ventilation in acute brain injury suggested considering at least prone position in patients who have concurrent ARDS and acute brain injury but no significant ICP elevation, whereas the role of RMs, CO_2_ removal systems, and ECMO is still uncertain [20, 52, 53].

Our findings suggest that each rescue therapy has specific effects on systemic and cerebral oxygenation, which reflect specific pathophysiological effects of each strategy on systemic and cerebral dynamics.

RMs and respiratory dialysis/ECCO_2_R seem to have no beneficial effect on systemic and cerebral oxygenation, whereas prone positioning and iNO can improve both systemic and cerebral oxygenation. Both RMs and prone positioning may increase ICP, but RMs seem to have a major effect on the hemodynamic system, causing an important reduction of MAP and thus reducing CPP. Prone positioning led to an increase in both the arterial and venous components of rSO_2_, suggesting an increase in CPP and a reduction of jugular venous return, causing only slightly increased nICP, whereas after RMs the arterial component was reduced (consequent to arterial hypotension) and the venous component was slightly increased (consequent to impairment of venous return), causing a substantial increase in ICP. iNO had no detrimental effect on MAP, nCPP, or nICP and should therefore be considered in cases of systemic and cerebral hypoxemia when ICP is unstable. Respiratory dialysis/ECCO_2_R has good efficacy in reducing both PaCO_2_ and nICP values, but it also causes a reduction in systemic and cerebral oxygenation. While reduction of rSO_2_ could be consequent to a rapid reduction of PaCO_2_, thus causing cerebral arterial vasoconstriction (with decrease in the arterial component of rSO_2_), the effect on systemic oxygenation is unclear; however, we hypothesize that the combination of higher pHa and lower PaCO_2_ reduces hypoxic pulmonary vasoconstriction, leading to lower oxygenation [58].

Overall, we found a strong correlation between systemic and cerebral oxygenation, thus confirming that systemic oxygenation values are the major determinants of cerebral oxygen status, as previously demonstrated [59–62]. Also, the strong correlation we found between the changes in SpO_2_ and rSO_2_ suggests that oxygenation should be monitored constantly during RMs, as changes in SpO_2_ are promptly reflected by changes in rSO_2_. Maneuvers that decrease systemic oxygen saturation expose the patient to the risk of cerebral hypoxia. As expected, rSO_2_ was also correlated to PaCO_2_ and MAP values, as both can be surrogates of cerebral blood flow and volume.

### Limitations

Several limitations of this study need to be mentioned. First, this is a single-center study with a small number of patients, especially in each subgroup; second, as this is an observational study, we only analyzed the rescue therapies currently adopted in our practice. For example, the type of RM adopted and the dose used for iNO test—although not completely established in the literature [63]—reflect our own policies.

Data on ECMO are missing as we opted to use ECCO_2_R to provide protective ventilation [64], with less need of external blood flow—minimizing the potential risks.

In this context, after ECCO_2_R PEEP from T0 and T1 was reduced. However, PEEP reduction was on average 1.5 cmH2O, clinically not significant and likely not affecting the results.

Moreover, we only evaluated the early effects of ventilator strategies on cerebral and systemic hemodynamics. Although we are aware that some rescue therapies might require time of application to produce a clinically relevant effect [65], we decided to focus on the early phase in order to evaluate the possible acute effects on cerebral oxygenation. As suggested by Chiumello et al. [66], PEEP variations exert their effects on oxygenation after a precise time lag, but in individual patients the change of oxygenation-related variables after PEEP modifications observed after 5′ can predict the changes observed after 60′.

Further, more specific data on physiological parameters including invasive neuro, respiratory, and hemodynamic monitoring would have been useful to assess changes in these parameters consequent to the application of rescue therapies. In particular, we assessed noninvasive ICP using TCD, using a formula which has been previously validated in experimental settings and brain-injured patients [67–69], but not in the general ICU population. However, although this method presents some limitations [70] in terms of accuracy, it has shown to be reliable to exclude intracranial hypertension and to assess the trajectory of ICP [71], making it very suitable in the context of our study. Also, we did not study patients’ autoregulatory status, which can also influence the rSO_2_ response to hypoxia. Finally, our population represents a specific subgroup with peculiar characteristics, and our results may thus not be generalizable to other clinical settings.

## Conclusions

In our population of COVID-19 patients with severe ARDS, ample physiologic variability was observed, with different early effects of rescue therapies on cerebral and systemic oxygenation. Treatment must be personalized and should take in consideration both pulmonary and cerebral needs. Strict use of neuromonitoring is warranted even in patients who are not primarily brain-injured, in order to prevent and ensure early detection of neurological complications. Future multicenter studies are warranted to confirm our results.

## Supplementary Information

**Additional file 1.** STROBE checklist, and additional analysis.

## Data Availability

Data are fully available upon request from the corresponding author.

## Abbreviations

ARDS: Acute respiratory distress syndrome
cHbi: The sum of ΔO_2_Hbi and ΔHHbi values
COVID-19: Coronavirus disease-2019
CPP: Cerebral perfusion pressure
CRRT: Continuous renal replacement therapy
Crs: Respiratory system compliance
CVVHD: Continuous venovenous hemodialysis
ECCO_2_R: Extracorporeal carbon dioxide removal
ECMO: Extracorporeal membrane oxygenation
FiO_2_: Fraction of inspired oxygen
FVd: Diastolic flow velocity
FVm: Mean flow velocity
FVs: Systolic flow velocity
Hb: Hemoglobin
HHbi: Index associated with changes in the deoxygenated component of Hb
iNO: Inhaled nitric oxide
MAP: Mean arterial pressure
MCA: Middle cerebral artery
nCPP: Noninvasive cerebral perfusion pressure
nICP: Noninvasive intracranial pressure
O_2_Hbi: Oxygenated component of Hb
PaCO_2_: Partial pressure of carbon dioxide
PaO_2_: Partial pressure of oxygen
PEEP: Positive end-expiratory pressure
Pplat: Plateau pressure
RMs: Recruitment maneuvers
RR: Respiratory rate
rSO_2_: Total value of regional cerebral oxygen saturation
SARS-CoV-2: Severe acute respiratory syndrome coronavirus-2
VT: Tidal volume
