# Supplementary material for: Early effects of ventilatory rescue therapies on systemic and cerebral oxygenation in mechanically ventilated COVID-19 patients with acute respiratory distress syndrome: a prospective observational study
Source: Crit Care. 2021 Mar 19;25:111. doi: 10.1186/s13054-021-03537-1 (PMC7978164; doi:10.1186/s13054-021-03537-1)
Supplement: Supplementary file 1 — Additional file 1. STROBE checklist, and additional analysis. [file 13054_2021_3537_MOESM1_ESM.docx]

**Supplementary material**

**Early Effects of ventilatory rescue therapies on systemic and cerebral oxygenation in mechanically ventilated COVID-19 patients with acute respiratory distress syndrome: a prospective observational study**

Chiara Robba^1,2^, Lorenzo Ball^1,2^, Denise Battaglini^2^, Danilo Cardim^3^,Emanuela Moncalvo^1^,Iole Brunetti^2^, Matteo Bassetti ^4,5^, Daniele R Giacobbe^5^, Antonio Vena^5^, Nicolo’ Patroniti^1,2^, Patricia RM Rocco^6^**,** Basil F Matta^7^, and Paolo Pelosi^1,2^ and collaborators*

^1^Department of Surgical Sciences and Integrated Diagnostics (DISC), University of Genoa, Italy

^2^Ospedale Policlinico San Martino, IRCCS for Oncology and Neuroscience, Genoa, Italy

^3^Department of Neurology, University of Texas, USA

^4^Department of Health Sciences (DISSAL), University of Genoa, Italy

^5^Infectious Diseases Unit, Ospedale Policlinico San Martino, IRCCS for Oncology and Neuroscience, Genoa, Italy

^6^Laboratory of Pulmonary Investigation, Carlos Chagas Filho Institute of Biophysics, Federal University of Rio de Janeiro, Rio De Janeiro, Brazil

^7^Neurocritical Care Unit, Addenbrooke’s Hospital, Cambridge, UK

**Table of contents**

Additional file 1. STROBE Statement—checklist of items that should be

included in reports of observational studies Page 2

Additional file 2. Figure S1. Example of noninvasive monitoring of cerebral Page 4

oxygenation during ECCO_2_R

Additional file 3. Figure S2. Scatterplots showing the correlation between Page 6

changes in rSO_2_ and changes in SpO_2_ and PaO_2_

Additional file 4. Table S1. Characteristics of patients included in the analysis Page 7

Additional file 5. Table S2. Subgroup analysis on the effect of rescue Page 8

therapies on cerebral and systemic variables according to patients with

low PaO_2_ (<66 mmHg) and high PaO_2_ (>or = 66mmHg)

Additional file 1. STROBE Statement—checklist of items that should be included in reports of observational studies.

|  | Item No | Recommendation | Page  No |
| --- | --- | --- | --- |
| **Title and abstract** | 1 | (*a*) Indicate the study’s design with a commonly used term in the title or the abstract | 1 |
|  |  | (*b*) Provide in the abstract an informative and balanced summary of what was done and what was found | 3,4 |
| Introduction | | | |
| Background/rationale | 2 | Explain the scientific background and rationale for the investigation being reported |  |
| Objectives | 3 | State specific objectives, including any prespecified hypotheses | 6 |
| Methods | | | |
| Study design | 4 | Present key elements of study design early in the paper | 6-7 |
| Setting | 5 | Describe the setting, locations, and relevant dates, including periods of recruitment, exposure, follow-up, and data collection | 7 |
| Participants | 6 | (*a*) *Cohort study*—Give the eligibility criteria, and the sources and methods of selection of participants. Describe methods of follow-up.  *Case-control study*—Give the eligibility criteria, and the sources and methods of case ascertainment and control selection. Give the rationale for the choice of cases and controls.  *Cross-sectional study*—Give the eligibility criteria, and the sources and methods of selection of participants | 7 |
|  |  | (*b*) *Cohort study*—For matched studies, give matching criteria and number of exposed and unexposed  *Case-control study*—For matched studies, give matching criteria and the number of controls per case |  |
| Variables | 7 | Clearly define all outcomes, exposures, predictors, potential confounders, and effect modifiers. Give diagnostic criteria, if applicable | 8-12 |
| Data sources/ measurement | 8* | For each variable of interest, give sources of data and details of methods of assessment (measurement). Describe comparability of assessment methods if there is more than one group | *12* |
| Bias | 9 | Describe any efforts to address potential sources of bias | 12 |
| Study size | 10 | Explain how the study size was arrived at | 12 |
| Quantitative variables | 11 | Explain how quantitative variables were handled in the analyses. If applicable, describe which groupings were chosen and why | 12 |
| Statistical methods | 12 | (*a*) Describe all statistical methods, including those used to control for confounding | 12 |
|  |  | (*b*) Describe any methods used to examine subgroups and interactions | 12 |
|  |  | (*c*) Explain how missing data were addressed | 12 |
|  |  | (*d*) *Cohort study*—If applicable, explain how loss to follow-up was addressed  *Case-control study*—If applicable, explain how matching of cases and controls was addressed  *Cross-sectional study*—If applicable, describe analytical methods taking account of sampling strategy | 12 |
|  |  | (*e*) Describe any sensitivity analyses |  |

| Results | | | |
| --- | --- | --- | --- |
| Participants | 13* | (a) Report numbers of individuals at each stage of study—eg numbers potentially eligible, examined for eligibility, confirmed eligible, included in the study, completing follow-up, and analysed | 13 |
|  |  | (b) Give reasons for non-participation at each stage |  |
|  |  | (c) Consider use of a flow diagram |  |
| Descriptive data | 14* | (a) Give characteristics of study participants (e.g. demographic, clinical, social) and information on exposures and potential confounders | 13 |
|  |  | (b) Indicate number of participants with missing data for each variable of interest | 14,15 |
|  |  | (c) *Cohort study*—Summarise follow-up time (e.g., average and total amount) | 14,15 |
| Outcome data | 15* | *Cohort study*—Report numbers of outcome events or summary measures over time | 14,15 |
|  |  | *Case-control study—*Report numbers in each exposure category, or summary measures of exposure | 13,14,15 |
|  |  | *Cross-sectional study—*Report numbers of outcome events or summary measures | 13,14,15 |
| Main results | 16 | (*a*) Give unadjusted estimates and, if applicable, confounder-adjusted estimates and their precision (e.g., 95% confidence interval). Make clear which confounders were adjusted for and why they were included | 13,14,15 |
|  |  | (*b*) Report category boundaries when continuous variables were categorized | 14,15 |
|  |  | (*c*) If relevant, consider translating estimates of relative risk into absolute risk for a meaningful time period | 14,15 |
| Other analyses | 17 | Report other analyses done—e.g. analyses of subgroups and interactions, and sensitivity analyses | 14,15 |
| Discussion | | | |
| Key results | 18 | Summarise key results with reference to study objectives | 16 |
| Limitations | 19 | Discuss limitations of the study, taking into account sources of potential bias or imprecision. Discuss both direction and magnitude of any potential bias | 19 |
| Interpretation | 20 | Give a cautious overall interpretation of results considering objectives, limitations, multiplicity of analyses, results from similar studies, and other relevant evidence | 16-17 |
| Generalisability | 21 | Discuss the generalisability (external validity) of the study results | 16-18 |
| Other information | | | |
| Funding | 22 | Give the source of funding and the role of the funders for the present study and, if applicable, for the original study on which the present article is based | 21 |

*Give information separately for cases and controls in case-control studies and, if applicable, for exposed and unexposed groups in cohort and cross-sectional studies.

**Note:** An Explanation and Elaboration article discusses each checklist item and gives methodological background and published examples of transparent reporting. The STROBE checklist is best used in conjunction with this article (freely available on the Web sites of PLoS Medicine at http://www.plosmedicine.org/, Annals of Internal Medicine at http://www.annals.org/, and Epidemiology at http://www.epidem.com/). Information on the STROBE Initiative is available at [www.strobe-statement.org](http://www.strobe-statement.org).

Additional file 2. Figure S1. Example of noninvasive monitoring of cerebral oxygenation during ECCO_2_R. Immediately after ECCO_2_R initiation, a reduction in rSO3 was observed, with particular reduction of the ΔO_2_Hbi component, probably consequent to CO_2_ reduction and cerebral arterial vasoconstriction.

**
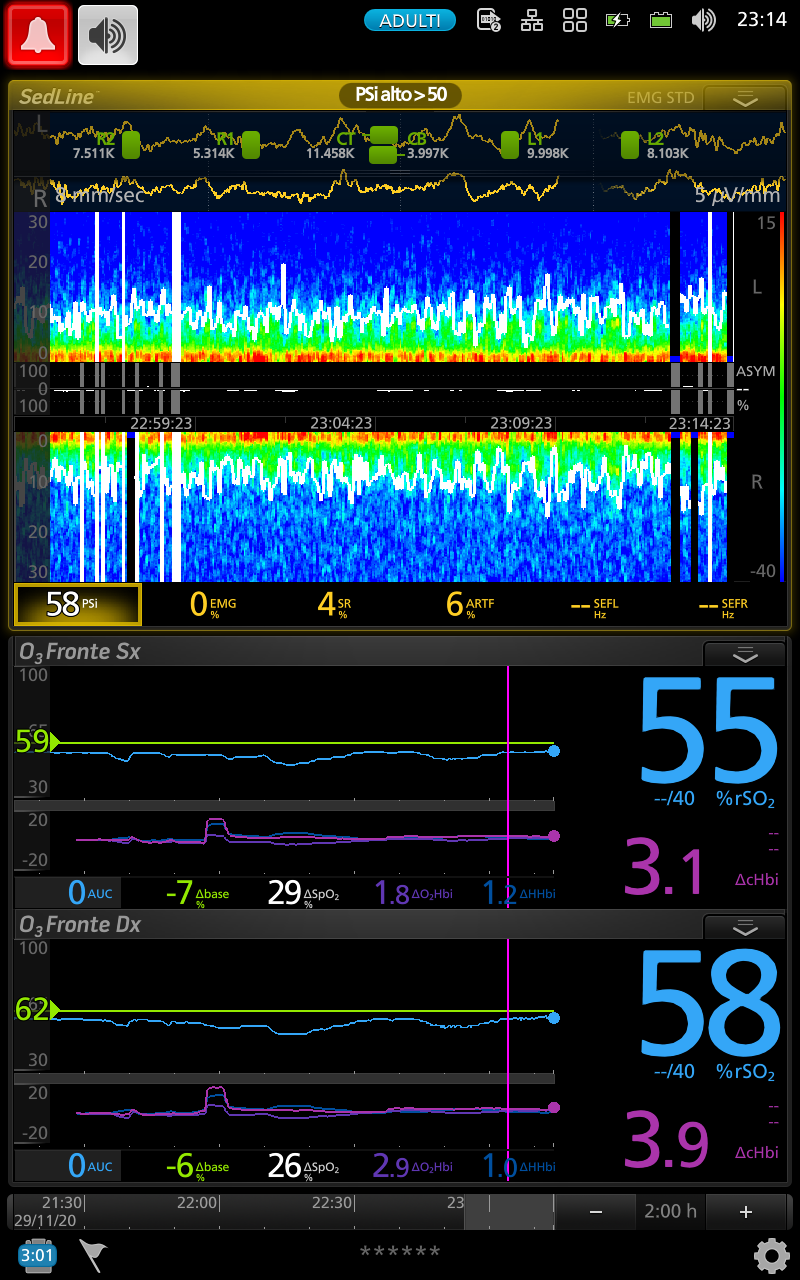
**

Additional file 3. Figure S2. Scatterplots showing the linear association and correlation (R) between changes in rSO_2_ and changes in SpO_2_ (plot A) and PaO_2_ (plot B). Repeated measurements for each patient are plotted in the same color pattern. Linear regression lines are correspondent to repeated measurements within patients.

**

**

Additional file 4. Table S1. Characteristics of patients included in the analysis

| **Characteristics of patients** | **All patients (n=22)** |
| --- | --- |
| Demographics  Gender, male [n, (%)]  Age [years], median [IQR]  BMI [kg/m^2^], median [IQR]  PBW [kg], median [IQR]  Comorbidities  Respiratory disease [n, (%)]  Cardiovascular disease [n, (%)]  Cancer [n, (%)]  Neurologic disorders [n, (%)]  Moderate/severe liver disease [n, (%)]  End-stage kidney injury [n, (%)]  Hypertension [n, (%)]  Diabetes mellitus [n, (%)] | 18 (81.8)  62 [57-68.5]  26 [24.7-29.2]  70[63.5-75]  3 (13.6)  5(50)  0 (0)  1 (4.5)  1 (4.5)  0 (0)  12 (54.5)  2 (9) |
| ICU characteristics at admission  SOFA score at admission, median [IQR]  PaO_2_/FiO_2_, median [IQR]  PEEP, median [IQR]  V_T_ [mL], median [IQR]  Pplat [cm/H_2_O], median [IQR]  Crs [mL/cmH_2_O], median [IQR]  D –dimer [ng/mL], median [IQR]  C-reactive protein [mg/dL], median [IQR]  Procalcitonin [ng/mL], median [IQR]  Interleukin-6 [pg/mL], median [IQR]  Creatinine [mg/dL], median [IQR]  Heart rate [n/min], median [IQR]  Mean arterial pressure [mmHg], median [IQR] | 5 [4-7.5]  81[65-82.5]  10[8.5-12.5]  418[360-530]  27[24.5-28.5]  23.5[22-29.5]  1996[1048.4-6888]  102[59.4-131]  0.74[0.19-1.79]  37.7[21.1-79.5]  0.7[0.6-1.1]  84[71-98]  77[73-88.5] |
| ICU discharge characteristics  Dead [n, (%)]  Alive [n, (%)] | 13 (59.1)  9 (40.9) |
| *Table Legend: IQR, interquartile range; n, number; BMI, body mass index; PBW, predicted body weight; SOFA, sequential organ failure assessment; ICU, intensive care unit; PaO_2_/FiO_2_, arterial partial pressure of oxygen/fraction of inspired oxygen; PEEP, positive end expiratory pressure; V_T_, tidal volume; Pplat, plateau pressure; Crs, respiratory system compliance.* | |

Additional file 5. Table S2. Subgroup analysis on the effect of rescue therapies on cerebral and systemic variables according to patients with low PaO_2_ (<66 mmHg) and high PaO_2_ (>or = 66mmHg) according to the median value of PaO2 in the overall population. Partial pressure of oxygen (PaO_2_), cerebral oxygenation (rSO2), oxygen saturation (SpO_2_), partial pressure of carbon dioxide (PaCO_2_), mean arterial pressure (MAP).

|  | **Low PaO2**  **(N=22)** | | | | | | **High PaO2**  **(N=23)** | | | | | |
| --- | --- | --- | --- | --- | --- | --- | --- | --- | --- | --- | --- | --- |
| **Parameter** | **T0** | **T1** | | | | **p-value** | **T0** | **T1** | | **p-value** | | |
| **Primary outcome** |  | | | | | |  | | | | | |
| rSO_2_ (%) | 53[51-56] | 53[50-59] | | | 0.210 | | 53[51.7-54.2] | | 53[49-59] | | 0.514 | |
| PaO_2_ (mmHg) | 62[59-64] | 69[67-74] | | | 0.001* | | 70[69.7-74.2] | | 74[67.5-77.5] | | 0.038* | |
| SpO_2_ (%) | 88[86-89] | 90.5[89-93] | | | 0.001* | | 89[88-91] | | 90[88.7-92.25] | | 0.047* | |
| PaCO2 (mmHg) | 55[49-71] | 53[48-67] | 0.015* | | | | 52 [46-74] | | 50[46-66] | | 0.002* |  |
| MAP (mmHg) | 72 [67-76] | 70[63[76] | | 0.170 | | | 73.5[68.7-78.2] | | 72[64-77.2] | | 0.035* |  |
